# Enterohaemorrhagic *Escherichia coli* inhibits recycling endosome function and trafficking of surface receptors

**DOI:** 10.1111/cmi.12319

**Published:** 2014-07-07

**Authors:** Abigail Clements, Charlotte A Stoneham, R Christopher D Furniss, Gad Frankel

**Affiliations:** MRC Centre for Molecular Bacteriology and Infection, Department of Life Sciences, Imperial CollegeLondon, UK

## Abstract

Enteropathogenic and enterohaemorrhagic *Escherichia coli* (EPEC/EHEC) manipulate many cell processes by injecting effector proteins from the bacteria into the host cell via a Type III secretion system. In this paper we report that the effector protein EspG disrupts recycling endosome function. In particular, we found that following transferrin binding and endocytosis EspG reduces recycling of the transferrin receptor (TfR), the prototypical recycling protein, from an intracellular location to the cell surface, resulting in an accumulation of TfR within the cell. The surface levels of three receptors [TfR, epidermal growth factor receptor (EGFR) and β1 integrin] were tested and found to be reduced dependent on EspG translocation. Furthermore, disruption of recycling endosome function and the reduced surface presentation of receptors was dependent on the previously reported RabGAP activity and ARF binding ability of EspG. This paper therefore supports the previous hypothesis that EspG acts as an enzyme scaffold perturbing cell signalling events, in this case altering recycling endosome function and cell surface receptor levels during infection.

## Introduction

Enteropathogenic *Escherichia coli* (EPEC) and enterohaemorrhagic *Escherichia coli* (EHEC) colonize the gut mucosa by translocating between 20 and 50 type III secretion system (T3SS) effectors into host cells to facilitate infection (Wong *et al*., 2011). The disruption of important cellular processes, including actin dynamics, immune signalling and apoptosis by T3SS effectors during EPEC/EHEC infection, has been well characterized (Wong *et al*., 2011). However, regulation of vesicle trafficking, which is critical for survival and replication of intracellular pathogens such as *Salmonella* (Ramsden *et al*., 2007), *Legionella* (Ge and Shao, 2011), *Shigella* and *Listeria* (Cossart and Roy, 2010), has not been well described for extracellular pathogens such as EPEC/EHEC.

Recently, a T3SS effector of EPEC/EHEC, EspG, which has a homologue in *Shigella* sp, VirA, was shown to act as a Rab GTPase activating protein (RabGAP); hydrolysing GTP to GDP to inactivate the Rab small GTPases (Dong *et al*., 2012). Interestingly, VirA showed low Rab specificity *in vitro* (active on 21 of the 30 mammalian Rabs tested) compared with EPEC EspG, which had increased specificity (active on only 8 of 30 Rabs). EspG has also been shown to interact with a number of other proteins including ARF GTPases (Selyunin *et al*., 2011; Dong *et al*., 2012), p21 activated kinases (Germane and Spiller, 2011; Selyunin *et al*., 2011), GM130, RACK1 (Clements *et al*., 2011) and tubulin (Hardwidge *et al*., 2005). The RabGAP activity of VirA has been shown to reduce autophagic recognition of intracellular *Shigella*, therefore protecting *Shigella* from degradation in the cytosol (Dong *et al*., 2012). In the same study, EPEC EspG was shown to reduce hGH secretion and IL-8 release. The phenotypes of both VirA and EspG were attributed specifically to Rab1 inactivation and the resultant decrease in ER-Golgi transport (Dong *et al*., 2012), and a model of EspG activity has been proposed where EspG interacts with ARF1 at the *cis*-Golgi membrane and deactivates the local Rab1 population resulting in bidirectional ER-Golgi trafficking arrest (Selyunin *et al*., 2014).

A recent study indicates that EspG is one of the most well conserved T3SS effectors across all EPEC/EHEC lineages (Hazen *et al*., 2013), pointing to an important role during infection. Disruption of protein secretion during EHEC infection has not been reported, however using hGH release (Dong *et al*., 2012) or secreted embryonic alkaline phosphatase (SEAP) (Kim *et al*., 2007) assays EPEC infection has been shown to disrupt protein secretion through both EspG and EspI/NleA, an additional T3SS effector which interacts with COPI vesicles to disrupt ER-Golgi trafficking (Kim *et al*., 2007). Considering that EHEC also carries EspG and EspI/NleA we hypothesized that it also has the ability to disrupt protein secretion. The aim of this study was to determine the impact of EspG on protein secretion and vesicle trafficking during EHEC infection, in order to understand how this might contribute to the overall infection strategy of EHEC.

## Results

### EHEC infection reduces protein secretion only upon EspG overexpression

**Figure 1:**
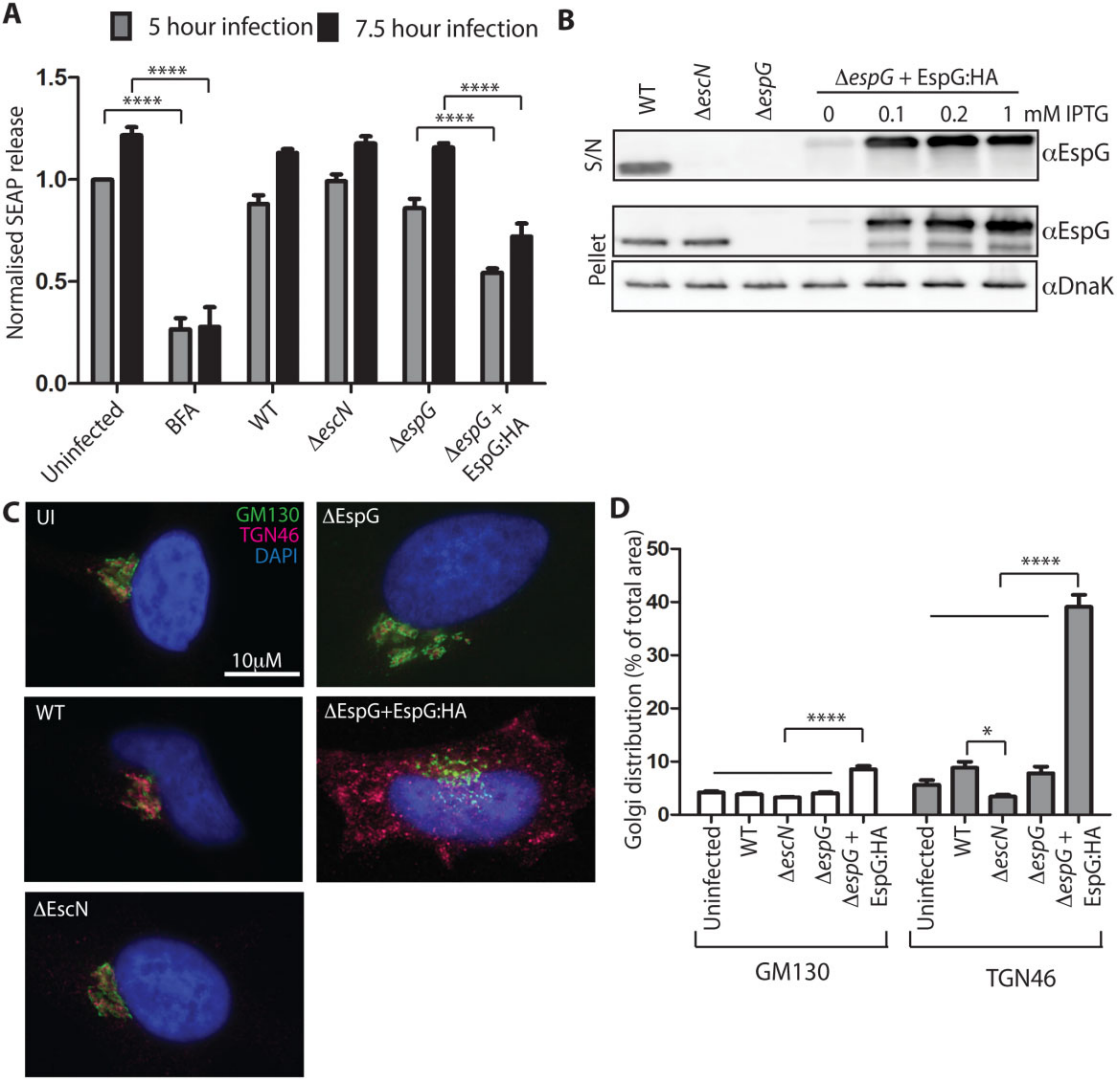
Wild type EHEC does not reduce SEAP secretion; overexpressed EspG reduces SEAP secretion and disrupts the Golgi. A. SEAP secretion was measured from HeLa cells expressing SEAP and infected for 5 or 7.5 h with the indicated EHEC strains. The percentage secreted SEAP was normalized to uninfected cells from the 5 h infection time-point. Results are mean ± SEM from three independent experiments. ****P < 0.0001. B. The expression (‘pellet’) and secretion (‘S/N’) of EspG was compared between the indicated EHEC strains grown under the conditions used for cell infection experiments. EspG and DnaK (loading control) were detected with specific antibodies to each. C. Golgi integrity was assessed by staining HeLa cells infected with the indicated EHEC strains for 5 h with GM130 (*cis*-Golgi marker) and TGN46 (*trans*-Golgi network marker) and D. *cis*-Golgi and TGN dispersion was quantified as a percentage of the total area of the cell using ImageJ (35–40 cells per condition). *P < 0.05, ****P < 0.0001.

We determined whether EHEC infection disrupted protein secretion by utilizing the SEAP assay as previously described (Kim *et al*., 2007; Clements *et al*., 2011). HeLa cells expressing SEAP were infected with wild type EHEC strain EDL933, a T3SS mutant (Δ*escN*) and Δ*espG* for 5 or 7.5 h. The protein secretion assay revealed that while a low level of secretion was seen from cells treated with brefeldin A (BFA), the infected cells secreted SEAP as effectively as uninfected control cells (Fig. 1A). The small, but reproducible difference between EDL933 and Δ*escN* infected cells may be due to EspI/NleA; however, no difference between EDL933 and Δ*espG* was seen. In contrast, reduced SEAP secretion was seen in cells infected with the complemented *espG* EHEC mutant (Δ*espG*+pEspG:HA), which overexpresses EspG, although the effect was not as pronounced as that seen following BFA treatment. This suggests that disruption of protein secretion by EspG requires a higher level of EspG than is translocated during WT infection conditions.

To compare the levels of EspG expressed and secreted in WT and Δ*espG*+pEspG:HA bacteria, immunoblots of the bacterial supernatant and pellet were probed with EspG antibodies (Fig. 1B). As expected EspG can be detected in the supernatant and pellet of WT bacteria, in the pellet only of Δ*escN* bacteria and in neither the pellet nor supernatant of the Δ*espG* bacteria. Uninduced Δ*espG*+pEspG:HA expressed and secreted barely detectable levels of EspG while all IPTG induced samples expressed and secreted much higher amounts of EspG than WT bacteria. Band intensity quantification with ImageJ indicates induced Δ*espG*+pEspG:HA secretes 2–3× the amount of EspG as WT.

### EspG overexpression alters the Golgi integrity

As ectopically expressed EspG localizes to and disrupts the Golgi structure and function (Clements *et al*., 2011; Selyunin *et al*., 2011; Dong *et al*., 2012), we investigated whether this also occurred during EHEC infection. While endogenous levels of EspG (i.e. WT EHEC infection) showed normal *cis*-Golgi and a slightly disrupted trans-Golgi network (TGN) staining patterns, the trans-complemented *espG* mutant significantly disrupted the *cis*-Golgi and the TGN (Fig. 1C). TGN staining was virtually indistinguishable from the *cis*-Golgi staining in uninfected cells, and cells infected with EHEC WT, Δ*escN* and Δ*espG* strains. In contrast in cells infected with EHEC Δ*espG*+pEspG:HA the TGN staining was discrete from the *cis*-Golgi structures, which were fragmented into smaller stacks. Quantification of the distribution of Golgi staining supports these conclusions. The distribution of the *cis*-Golgi marker (GM130) was indistinguishable in all infected populations except the cells infected with EHEC Δ*espG*+pEspG:HA. In this population the distribution of the *cis*-Golgi staining doubled (8.6 ± 0.59% of total cell area compared with 4.2 ± 0.23% of total cell area) for uninfected cells). The increased distribution of the *trans*-Golgi network staining in cells infected with EHEC Δ*espG*+pEspG:HA was even more pronounced (39.1 ± 2.3% of total cell area compared with 5.6 ± 0.9% of total cell area for uninfected cells). WT infected cells showed a small but insignificant increase in TGN distribution compared with uninfected or Δ*escN* infected cells. These results indicate that high levels of EspG expression can disrupt Golgi morphology and reduce SEAP secretion (Fig. 1A), consistent with previous findings using ectopically expressed EspG (Clements *et al*., 2011; Selyunin *et al*., 2011; Dong *et al*., 2012).

### EspG localizes with TGN and RE markers

**Figure 2:**
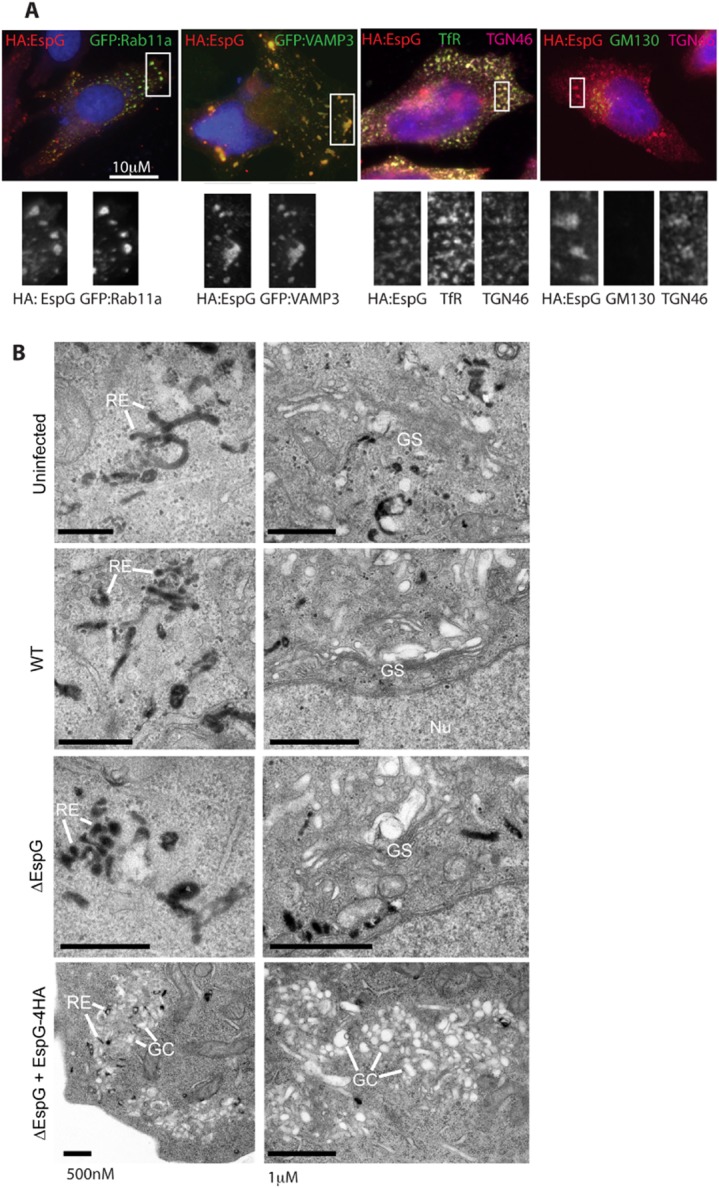
EspG localizes to recycling endosomes and the trans-Golgi network, altering their morphology. A. Localization of EspG:HA was characterized by immunofluorescence analysis of recycling endosome markers (GFP:Rab11a, GFP:VAMP3 and endogenous TfR staining) or Golgi markers (endogenous TGN46 and GM130 staining). All cells were infected with Δ*espG* + pEspG:HA for 5 h and co-stained with DAPI and HA, representative images are shown. B. TEM analysis of uninfected or infected cells incubated with Tf-HRP before fixation and DAB staining to reveal electron-dense Tf-positive vesicles. Representative images of the distribution of cellular organelles, including tubular recycling endosomes (RE), Golgi stacks (GS) or Golgi compartments (GC) are indicated.

In order to understand the function of EspG during infection we visualized its cellular localization. However, we were unable to detect endogenous levels of EspG by immunofluorescence and therefore used the trans-complemented *espG* mutant expressing HA tagged EspG (Δ*espG*+pEspG:HA). EspG:HA staining coincided with markers of recycling endosomes (REs) Rab11a, transferrin receptor (TfR) and VAMP3; and the TGN (TGN46) (Fig. 2A). No colocalization was seen with the *cis*-Golgi marker GM130.

To allow closer investigation of the endomembrane system, cells were analysed by transmission electron microscopy (TEM) using horseradish peroxidise (HRP)-conjugated holo-Tf to identify the Tf-positive endosomes (recycling and early endosomes). The TfR is a prototypical recycling surface protein, which specifically binds Tf in its iron-bound form (holo-Tf) at the plasma membrane. Following binding the TfR:holo-Tf complex is rapidly endocytosed. Iron is released in the sorting endosomes, while Tf remains bound to its receptor for transport back to the cell surface where the neutral extracellular pH promotes release of iron-free Tf. Tf:TfR recycling may occur either directly from the sorting endosome (fast recycling), or via RE (Maxfield and McGraw, 2004). Recycling compartments were identified by TEM analysis of uninfected cells, as clusters of Tf-positive endosomes of tubular morphology with both peripheral and pericentriolar distribution (Fig. 2B, uninfected, labelled RE). Tf-positive tubular endosomes in cells infected with EHEC WT, Δ*espG* appeared of similar morphology (Fig. 2B). In contrast, overexpression of EspG (Δ*espG* + pEspG:HA) was associated with peripherally located accumulations of Tf-positive tubular endosomes, closely associated with electron-lucent vesicles, reminiscent of Golgi compartments (GC). This phenotype was associated with a lack of discernible juxtanuclear Golgi stacks (GS), which were clearly identifiable in all other infection conditions tested. This suggests that the accumulation of EspG:HA staining observed by immunofluorescence studies is due to formation of heterologous aggregates of REs and Golgi components.

### Endogenous EspG reduces surface levels of TfR

**Figure 3:**
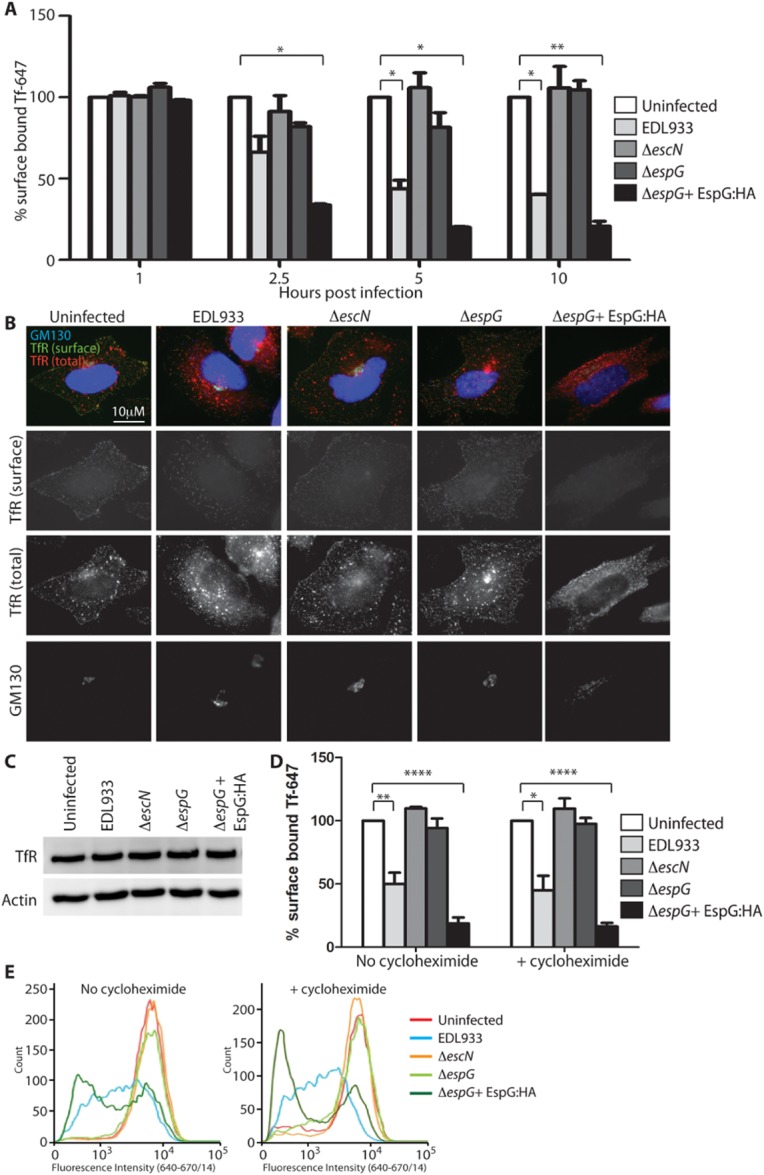
EspG reduces the amount of surface-localized TfR. A. Surface-localized TfR on HeLa cells infected with the indicated EHEC strains was assessed by addition of Tf-647 at the indicated time-points post infection. The median fluorescence intensity (MFI) of surface-bound Tf-647 is expressed as a percentage of the MFI of uninfected cells. Results are expressed as the mean ± SEM of two independent experiments. *P < 0.05; **P < 0.01. B. Localization of the TfR was determined by immunofluorescence of infected HeLa cells before (TfR surface) and after (TfR total) permeabilization.C. Total TfR levels in all infected cell lysates was detected by immunoblot (actin serves as a loading control). D and E. (D) Surface-localized TfR was assessed on HeLa cells infected for 5 h with and without cycloheximide (20 μg ml^−1^) treatment throughout the infection to inhibit new TfR synthesis. The MFI of surface-bound Tf-647 is expressed as a percentage of the MFI of uninfected cells and the mean ± SEM of three independent experiments plotted and representative histograms of fluorescence intensity shown in (E). *P < 0.05; **P < 0.01; ****P < 0.0001.

The observed localization of EspG with markers of REs led us to question whether receptor recycling was disrupted in infected cells. For this we continued using the TfR as a model recycling receptor and analysed the amount of TfR that was present at the cell surface over an infection time-course, by adding fluorescently labelled Tf to infected cells (Fig. 3A). We found that cells infected with WT EHEC (expressing endogenous EspG) bound less Tf on their cell surface from 5 h post infection compared with uninfected cells. Cells infected with either the Δ*escN* or Δ*espG* EHEC mutants bound equivalent Tf on the cell surface throughout the infection time-course, indicating that EspG was the only T3SS effector involved in this process. Cells infected with the complemented Δ*espG* mutant (Δ*espG* + pEspG:HA) exhibited reduced Tf-binding at an earlier time-point than WT EHEC (from 2.5 h post infection), however by 10 h post infection both WT EHEC and EHEC Δ*espG* + pEspG:HA retained less than 50% of the amount of Tf on the cell surface as uninfected cells or cells infected with EHEC Δ*espG* mutant. Therefore the surface localization of the TfR appears to be reduced dependent on EspG translocation. While overexpression of EspG was more effective in reducing surface localization of TfR, reduced surface TfR could be detected 5 h after infection with WT EHEC expressing endogenous levels of EspG. This is the first phenotype that can be clearly attributed to endogenous levels of EspG during EHEC infection.

We next established where the TfR was accumulating during EHEC infections. We used immunofluorescence staining to visualize total and surface-localized endogenous TfR (i.e. with and without cell permeabilization) (Fig. 3B). Following infection with WT EHEC the TfR did not accumulate in the ER or the Golgi, which would have been expected if secretion was being blocked. Instead, the TfR was observed accumulating in vesicles, which were more pronounced in cells infected with the strains translocating EspG (WT or Δ*espG* + pEspG:HA).

To confirm that the observed quantitative changes in surface TfR were not due to altered expression levels of the receptor, immunoblots were performed on total cell extracts. This revealed equivalent amounts of TfR in all the infected cell populations (Fig. 3C).

In order to further study the effect of EspG on the existing Tf pool, we repeated the surface labelling of the TfR with Tf-647 in the presence of cycloheximide which inhibits protein synthesis (Fig. 3D). A 5 h infection time-point was chosen for analysis. Inhibition of protein synthesis reduced the total amount of TfR detectable on the cell surface in all cell populations. Cells containing EspG (i.e. WT or Δ*espG* + pEspG:HA) further reduced the surface-localized TfR compared with uninfected cells or those infected with Δ*escN* or Δ*espG*. Collectively, these results show that endogenous levels of EspG can reduce the amount of TfR on the cell surface without altering TfR synthesis or transport of newly synthesized TfR. This suggests that EspG affects recycling of the existing population of TfR.

### EspG disrupts TfR recycling

**Figure 4:**
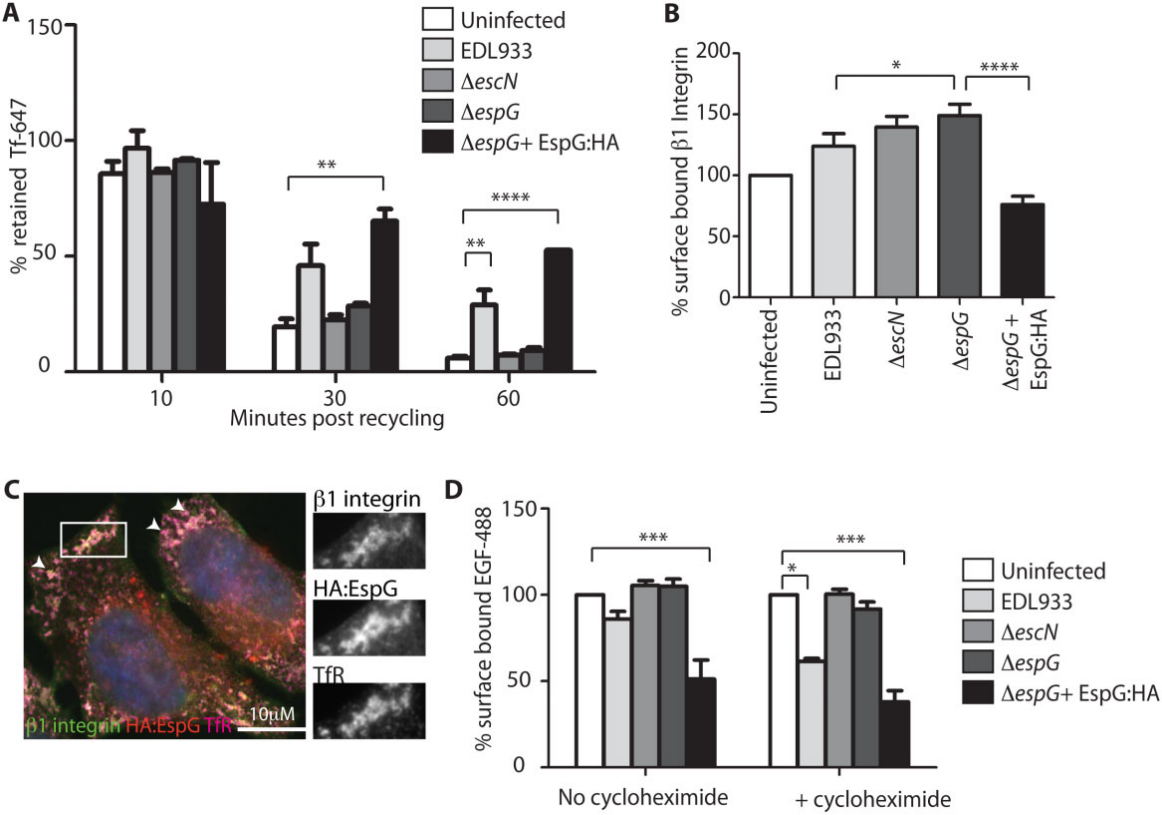
EspG reduces the recycling of TfR and the surface localization of β1 integrin and EGFR. HeLa cells infected for 5 h with the indicated EHEC strains were assessed in the following ways. A. TfR recycling was measured by comparing the cell-associated fluorescence of cells which have endocytosed fluorescent Tf to those that have undergone an unlabelled Tf chase for 10, 30 and 60 min. Data are expressed as ‘% retained Tf’ (Tf retained following chase/total Tf endocytosed). **P < 0.01; ****P < 0.0001. B. Surface-localized β1 integrin on EHEC-infected cells was quantified by indirect antibody staining of non-permeabilized cells and flow cytometry. The MFI is expressed as a percentage of the MFI of uninfected cells and the mean ± SEM of three independent experiments plotted. *P < 0.05; ****P < 0.0001. C. Fluorescence microscopy of cells infected with Δ*espG* + pEspG:HA and stained for β1 integrin, TfR and HA:EspG. Areas of co-staining are enlarged in single channel images and highlighted by arrows in merged image. D. Surface-localized EGFR was quantified by addition of EGF-488 to infected cells followed by quantification by flow cytometry. Cells were infected in the absence and presence of cycloheximide (20 μg ml^−1^). The MFI of surface-bound EGF-488 is expressed as a percentage of the MFI of uninfected cells and the mean ± SEM of three independent experiments plotted. *P < 0.05; ***P < 0.001; ****P < 0.0001.

The effect of EspG on recycling of the TfR was directly tested by chasing endocytosed fluorescent Tf with excess unlabelled Tf. As expected, in uninfected control cells the majority of fluorescent Tf was recycled from the cell over the 60 min time-course (Fig. 4A). A similar phenotype was seen in cells infected for 5 h with either the Δ*escN* or Δ*espG* EHEC mutants, indicating that in these cells recycling was occurring efficiently. In contrast, cells infected with WT EHEC or EHEC Δ*espG* + pEspG:HA retained over 30% of the endocytosed fluorescent Tf after 60 min of recycling, indicating that the recycling of the Tf/TfR complex was significantly impeded. Importantly, endogenous levels of EspG were sufficient for this pronounced phenotype to be observed. EspG therefore reduces surface TfR levels through disrupting recycling of the TfR.

### EspG reduces surface localization of β1 integrin and epidermal growth factor receptor (EGFR)

To determine if there was a general reduction of cell surface-localized receptors or whether this phenomenon was confined to the TfR, two additional surface receptors were analysed: β1 integrin and EGFR. β1 integrin is endocytosed in a clathrin and dynamin dependent process and recycled back to the membrane mainly via the slow (Rab11a), or tubular actin dependent recycling pathways (Roberts *et al*., 2004; Li *et al*., 2005). The amount of β1 integrin on the cell surface was determined by indirect staining of non-permeabilized infected cells (Fig. 4B). Interestingly cells infected with either EHEC Δ*escN* or Δ*espG* mutant had increased surface levels of β1 integrin compared with uninfected cells. This may be due to the presence of the bacterial outer membrane adhesin intimin, which binds, and therefore potentially stabilizes, surface β1 integrins (Frankel *et al*., 1996; Sinclair *et al*., 2006). Supporting the results from the TfR analysis, EspG expression (WT or Δ*espG* + pEspG:HA) also led to decreased surface levels of β1 integrin compared with those infected with EHEC Δ*espG*. EspG:HA staining of permeabilized cells indicated EspG was colocalizing with intracellular β1 integrin (Fig. 4C) and TfR. This again suggests EspG is impeding the recycling of β1 integrin back to the plasma membrane.

EGFR is internalized mainly by clathrin-mediated endocytosis into early endosomes where it is sorted into either late endosomes/lysosomes for degradation or recycled back to the plasma membrane by fast or slow pathways (Tomas *et al*., 2014). Cell surface levels of EGFR are therefore a combination of the rates of endocytosis, degradation, recycling and synthesis. Surface levels of EGFR were measured by addition of fluorescently labelled EGF (EGF-488 complex) to infected cells (Fig. 4D). Consistent with the TfR and β1 integrin results, less EGF-488 binding was detected on the surface of WT infected cells compared with uninfected cells or cells infected with Δ*escN* or Δ*espG* mutants. Again this difference was more pronounced in Δ*espG* + pEspG:HA infected cells. While these differences could be observed in the absence of cycloheximide (Fig. 4D, no cycloheximide) the effect of endogenous levels of EspG became more pronounced when cycloheximide was added (Fig. 4D, +cycloheximide), i.e. when the contribution of EGFR synthesis was removed from the assay. This again supports the finding that EspG alters cell surface receptors by inhibiting the function of recycling endosomes rather than altering protein synthesis and transport. When present at high levels (Δ*espG* + pEspG:HA) EspG both blocks recycling and protein secretion.

### EspG RabGAP and ARF binding activity are required to reduce surface TfR and EGFR levels

**Figure 5:**
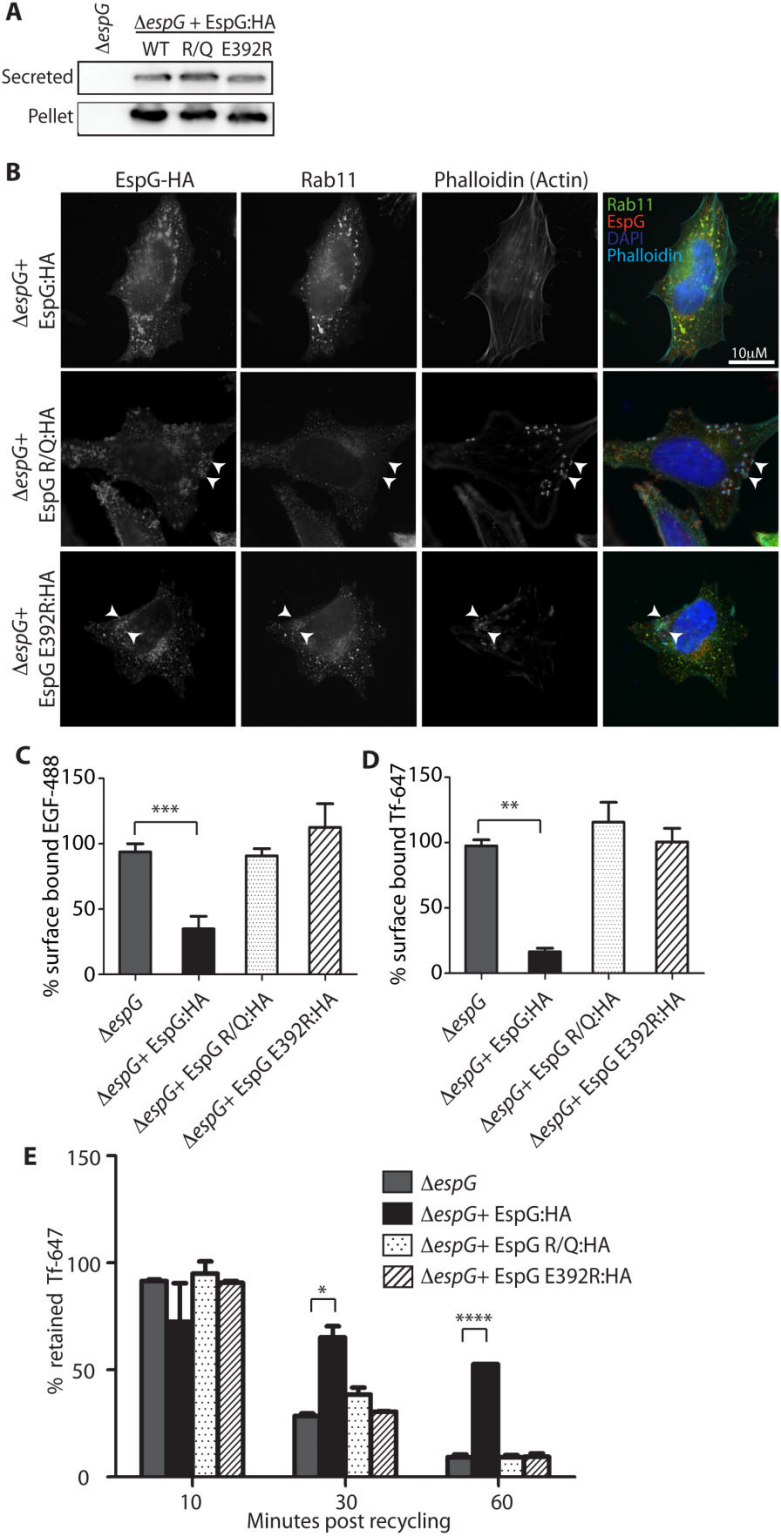
RabGAP activity and ARF binding ability are required for EspG activity. A. Expression and secretion of EspG-R/Q:HA (RabGAP deficient) and EspG-E392R:HA (ARF binding deficient) proteins from Δ*espG* compared with EspG:HA. B. Representative immunofluorescence images of the cellular localization of both complementation constructs compared with EspG:HA. Rab11–GFP expressing cells were infected for 5 h and stained with anti-HA antibodies and Phalloidin was used to detect actin pedestals which form under attached bacteria. C and D. Surface levels of (C) TfR or (D) EGFR of cells infected for 5 h with the indicated strains in the presence of cycloheximide (20 μg ml^−1^) were measured by addition of fluorescent ligands and quantified by flow cytometry. The MFI of surface-bound Tf-647 or EGF-488 is expressed as a percentage of the MFI of uninfected cells and the mean ± SEM of three independent experiments plotted.E. TfR recycling was measured by comparing the cell-associated fluorescence of cells which have endocytosed fluorescent Tf to those that have undergone unlabelled Tf chase for 10, 30 and 60 min. Data are expressed as ‘% retained Tf’ (Tf retained following chase/total Tf endocytosed). *P < 0.05; **P < 0.01; ***P < 0.001; ****P < 0.0001.

Defined EspG mutants which lack RabGAP activity (EspG-R/Q:HA) (Dong *et al*., 2012) or cannot bind ARFs (EspG-E392R:HA) (Selyunin *et al*., 2011) were created and tested for their ability to complement Δ*espG*. After confirming that both modified EspG proteins could be secreted at equivalent levels to Δ*espG* + pEspG:HA (Fig. 5A) we investigated their cellular localization by immunofluorescence (Fig. 5B). Again EspG:HA was clearly detected with discrete cytoplasmic vesicles that are removed from the attached bacteria and also contain Rab11–GFP. In contrast, EspG-R/Q:HA and EspG-E392R:HA display different cellular localizations. EspG-R/Q:HA staining was more dispersed with no punctuate staining at the bacteria or in discrete cytoplasmic vesicles, although enhanced staining could be observed surrounding attached bacteria. EspG-E392R:HA staining could be observed with Rab11 positive vesicles or as punctuate staining closely associated with an attached bacteria suggesting trafficking to its correct cellular localization may be impaired.

The ability of these mutants to complement Δ*espG* in functional assays was tested. While complementation with pEspG:HA reduces binding of Tf-647 and EGF-488 indicating reduced levels of surface TfR and EGFR, complementation with pEspG-R/Q:HA or pEspG-E392R:HA was unable to reduce binding of either ligand (Fig. 5C and D). Consistently neither mutant could complement the Δ*espG* mutant by inhibiting the recycling of the TfR (Fig. 5E). This suggests both the ARF binding ability and RabGAP activity of EspG are required for EspG to disrupt recycling endosomes and subsequently reduce the level of surface receptors.

## Discussion

Many bacterial pathogens inject multiple T3SS effector proteins over a short period of time, which manipulate a wide variety of host cell processes. The function of each individual effector during infection can be difficult to determine because of redundancy, cooperative or antagonistic effects. In this study we have shown that endogenous levels of EspG from the extracellular pathogen EHEC can drastically reduce the recycling of cargo through REs, resulting in reduced surface localization of receptors. To our knowledge this is the first example of specific interference in recycling pathways by any EPEC/EHEC T3SS effector.

During EPEC infection EspG has been reported to reduce protein secretion (Dong *et al*., 2012) however during EHEC infection this only became apparent when EspG was overexpressed. This could be due to the fact that EPEC, which adheres to cells more efficiently and encodes two EspG genes, might translocate more total EspG than EHEC. Alternatively EHEC EspG may have different ARF or Rab specificity to EPEC EspG, although the similarity in sequences makes this unlikely. We found that while endogenous levels of EspG were not able to reduce protein secretion they were sufficient to alter the function of REs, suggesting this is the primary effect of EspG during infection. EspG:HA localized with markers of RE and the TGN, two closely entwined endomembrane compartments. REs are a collection of sorting endosome-derived, microtubule-associated tubular vesicles which transport proteins and lipids to the plasma membrane (Maxfield and McGraw, 2004). The TGN is a major sorting centre of lipids and proteins and intersects the biosynthetic and endocytic pathways receiving and delivering cargo to (among others) REs (De Matteis and Luini, 2008). The continuous nature of the endomembrane system means that disruption of one component will have downstream effects upon others, and therefore disrupted secretion upon EspG overexpression may be a secondary effect of disrupted recycling during EHEC infection.

The molecular interactions of EspG during infection remain unclear. The ability of EspG to reduce receptor recycling requires both the RabGAP and ARF binding sites. The E392R ARF binding mutation also inhibits binding of EspG to PAKs *in vitro* (Selyunin *et al*., 2011) and therefore the inability of this mutant to complement Δ*espG* may be due to a combination of these factors. The cellular localization of the two mutant EspGs suggests that ARF binding and RabGAP activity may be required for the correct localization of EspG in REs as well as the correct functioning of the protein. Further work is necessary to understand the contribution of these factors during infection. *In vitro* EspG can interact with multiple Rabs and ARFs. Recently, Selyunin *et al*. proposed a mechanistic model for EspG interaction with Arf1/Rab1 in which EspG localizes to the ER/Golgi interface via Arf1, allowing targeted, local Rab1 GTP hydrolysis and the loss of Rab-dependent vesicle fusion at this compartment (Selyunin *et al*., 2014). As the strongest Arf interaction for EspG has been shown to be with Arf6 (Selyunin *et al*., 2011) and the specificity of EspG RabGAP activity is proposed to be determined by correct membrane targeting via ARF binding (Dong *et al*., 2012; Selyunin *et al*., 2014), we hypothesize that during infection EspG is sequestered by ARF6 at endosomal compartments, spatially restricting which Rabs EspG can inactivate. The specificity of EspG mediated GTP hydrolysis then further restricts which Rabs are inactivated by EspG. Some Rabs described to localize to recycling endosomes (Grant and Donaldson, 2009) are insensitive to EspG-mediated GTP hydrolysis (e.g. Rab 11 and 22) while others are sensitive (e.g. Rab 35) or untested (e.g. Rabs 8 and 10) (Dong *et al*., 2012). These latter groups may prove to be the actual targets of EspG during infection.

REs have many functions within the cell. Here we have investigated the best-described activity, the recycling of receptors from early endosomes back to the plasma membrane. Additional functions of recycling endosomes may also be impeded by EspG. For example the membrane required for autophagosome formation has been described to egress from recycling endosomes, where fusion of two major sources of autophagosome membranes (mAtg9 and ATG16L1-containing vesicles) occurs in a VAMP3 dependent process (Puri *et al*., 2013). The *Shigella* homologue VirA was shown to reduce the level of autophagosome formation around intracellular bacteria (Dong *et al*., 2012) and therefore the disruption of recycling endosome maturation may also contribute to the reduced autophagosome formation observed in the presence of VirA.

REs are also of great importance in polarized cells, which have multiple recycling endosome compartments (apical, and common) and correct segregation and trafficking of proteins through the REs is essential for establishing cell polarity and maintaining tight junctions (TJ) and adherens junctions (AJ). EspG has previously been shown to reduce transepithelial resistance (TER) during EPEC infection indicating an effect on TJ integrity (Tomson *et al*., 2005). EspG has also been reported to alter the localization of the major apical anion exchanger DRA from the plasma membrane to intracellular structures (Gill *et al*., 2007). Both of these phenotypes could be explained by the disruption of REs. The mis-localization of DRA results in altered chloride transport potentially contributing to the diarrhoea associated with EPEC/EHEC infection. This observation suggests a direct consequence of EspG disruption of REs on disease pathology. In addition to TJ/AJ proteins and ion channel transporters most cell surface receptors, including those responsible for the host response to infection, are also recycled. Further studies are required to determine the extent of cell surface changes that occur dependent on EspG and how this affects the infection dynamics of EPEC/EHEC. The realization that EspG subverts REs expands the extensive activities of T3SS effectors in bacterial pathogens as well as adding another fascinating tier to our understanding of EHEC and EPEC infection.

## Experimental procedures

### Bacterial strains and infections

*espG* was deleted from EHEC strain EDL933 using the lambda red system (Datsenko and Wanner, 2000) to generate strain Δ*espG* (ICC1074). EspG was cloned into pSA10 with a C-terminal 4xHA fusion to give pEspG:HA (pICC1391). pEspG-R/Q:HA (pICC1392) and pEspG-E392R:HA (pICC1393) were created by site-directed mutagenesis (sequential site-directed mutations of R208K then Q293A for pEspG-R/Q:HA). HeLa cells were maintained in DMEM (1 g l^−1^ glucose, 1 mM glutamax and 10% FCS) and seeded as appropriate for each assay. Thirty minutes prior to infection cells were washed and FCS-free DMEM (1 g l^−1^ glucose) added. Stationary phase EHEC cultures were diluted 1:1000 into DMEM (1 g l^−1^ glucose) and incubated stationary for 16–18 h, 37°C, 5% CO_2_. When required 0.1 mM IPTG was added to bacterial cultures 30 min prior to infection. EHEC cultures were diluted in DMEM (1 g l^−1^ glucose) and added to cells to give an MOI of approximately 100:1. Infected cells were centrifuged for 5 min at 500 *g* to synchronize attachment. After 2.5 h infected cells were washed with PBS, DMEM (1 g l^−1^ glucose, 100 μg ml^−1^ gentamicin) was added, and cells were incubated for a further 2.5 h unless indicated. When required cells were transfected 24 h prior to infection using Genejuice (Merck) according to manufacturers instructions.

### SEAP assay

HeLa cells were transfected with pSEAP2-Control and after 8 h seeded into 96 well plates at 2 × 10^4^ cells per well. Twenty-four hours post transfection cells were infected for 2.5 h as described above (Δ*espG* + pEspG:HA induced with 0.1 mM IPTG), washed and DMEM [no phenol Red, gentamicin (100 μg ml^−1^)] added for 2.5 or 5 h. Supernatant and cells were then assayed for SEAP as previously described (Clements *et al*., 2011). The percentage of SEAP released 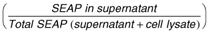 was calculated for each condition and then normalized to uninfected cells from the 5 h infection time-point. Results are mean ± SEM of three independent experiments.

### Immunofluorescence microscopy

HeLa cells were seeded in 24 well plates with glass coverslips 48 h prior to infection at 7.5 × 10^4^ cells per well in DMEM (1 g l^−1^ glucose, 1 mM glutamax and 10% FCS). Following infection for the appropriate times cells were washed 3× with PBS and fixed with 4% PFA for 15 min at room temperature (RT). PFA was removed, cells washed again with PBS (×3), neutralized with 50 mM NH_4_Cl for 15 min at RT, before permeabilization for 8 min with 0.05% Triton X-100 (when required). Cells were again washed with PBS (×3), blocked with 3% BSA in PBS for 30 min and stained with the appropriate antibodies in 1% BSA/PBS: mouse anti-GM130 (1:500, BD Biosciences), rabbit anti-giantin (1:500, Abcam), mouse anti-myc, clone 4A6 (1:500, Millipore), mouse anti-HA, clone 16B12 (1:500, Cambridge Biosciences), mouse anti-HA:TRITC, clone HA-7 (1:100, Sigma), rabbit anti-TfR (1:200, Millipore) sheep anti-TGN46 (1:300, Serotec) and goat anti-O157 (1:200, Fitzgerald). Primary antibodies were washed with PBS (×3) and secondary anti-IgG conjugates (AF488, RRX and Cy5, 1:200, Jackson Immunoresearch) in 1% BSA/PBS added for 45 min. DNA were stained with DAPI (1:1000, Invitrogen) and actin with Phalloidin conjugates (Sigma). Coverslips were washed with PBS (×3) and mounted using ProLong Gold Antifade mounting media (Life Technologies).

A widefield epifluorescence microscope with 100× oil objective (Axio Observer Z1) or a confocal microscope with 63× oil objective (Leica SP5) were used for visualization. Axio images were deconvolved using the Nearest Neighbour algorithm in AxioVision (Zeiss) and the slice of interest projected to form the new image. Golgi distribution was quantified in ImageJ by automatic thresholding of Golgi (either GM130 or TGN46) staining and measuring the % area covered for each cell.

### Transmission electron microscopy

Infected HeLa cells were washed with PBS (3×) and cooled on ice before fixation with 0.5% glutaraldehyde (Agar Scientific) in 200 mM sodium cacodylate (TAAB) for 5 min on ice, then at RT for a further 25 min. Cells were immediately washed in cacodylate buffer and Tf-HRP reacted with diaminobenzidine (DAB) in stable peroxide buffer (Metal Enhanced DAB Substrate Kit, Thermo Scientific) for 30 min at RT. The reaction was stopped by washing in sodium cacodylate before post-fixation in 1% osmium tetroxide/1.5% potassium ferrocyanide for 1 h at RT. The cells were then washed in ddH_2_O, stained overnight at 4°C with 0.5% uranyl acetate, washed with ddH_2_O and serially dehydrated in graded ethanol before infiltration with Epon 812 resin. Ultrathin sections (∼70 nm) of the flat-embedded cell monolayers were cut parallel to the surface of the dish, collected onto formvar-coated 50 mesh EM grids, and stained for 30 s with Reynolds' lead citrate before imaging. TEM samples were viewed by using an FEI Tecnai G^2^ electron microscope with a Soft Imaging System Megaview III charged-coupled-device camera. Images were collected at 1376 by 1032 by 16 pixels using AnalySIS version Docu software (Olympus Soft Imaging Solutions).

### Tf and EGF assays

HeLa cells were seeded in 12-well plates 24 h prior to infection at 2 × 10^5^ cells per well. Cycloheximide (20 μg ml^−1^) was added throughout the infection period when indicated. Following infection, surface-bound receptors were assessed by washing cells with ice-cold PBS then, on ice, adding 10 μg ml^−1^ Tf-647 (Invitrogen) or 2 μg ml^−1^ epidermal growth factor (EGF)-488 complex (Invitrogen) in DMEM (no FCS) for 30 min. Unbound Tf-647 or EGF-488 was removed, cells trypsinized (0.025% trypsin/0.02% EDTA), neutralized (DMEM + 10% FCS) and fixed in 4% PFA and stored in PBS for flow cytometry analysis on a BD LSR Fortessa.

For recycling assays, infected cells were washed and incubated with 10 μg ml^−1^ Tf-647 in DMEM (no FCS) for 60 min at 37°C. Cells were either processed for endocytosed Tf by washing with PBS, then trypsinized, neutralized, fixed and stored as above or recycling was measured by washing cells 2 × 5 min with 100 μg ml^−1^ unlabelled Tf in PBS on ice, then incubating with 100 μg ml^−1^ unlabelled Tf in DMEM for 10, 30 or 60 min at 37°C. Cells were then processed as for endocytosed Tf.

### Surface β1 integrin analysis

Infected cells were washed with PBS, detached using Cellstripper (Cellgro) and neutralized with DMEM +10% FCS. On ice, cells were blocked (2% FCS/PBS, 10 min), incubated with primary antibody for 45 min (0.25 μg per sample, β1 integrin, DHSB), washed and incubated with secondary conjugate for 30 min (0.5 μg per sample, rabbit anti-mouse IgG Fc, Jackson Immunoresearch). Cells were washed and fixed as above.

### Flow cytometry analysis

All samples were completed in triplicate and analysis performed on a BD LSR Fortessa on three independent occasions. A total of 10 000 cells were collected per sample and analysis performed using FlowJo software (Tree Star Inc.). The median fluorescence intensity (MFI) of gated live cells was calculated for each sample and the average of the triplicate samples determined.

### Immunoblotting

HeLa cells infected for 5 h were washed gently and lysed with 2× Laemmli Buffer. Samples were collected, boiled for 5 min, separated by 10% SDS-PAGE and transferred to PVDF. The membrane was cut in two and the TfR detected with rabbit anti-TfR (1:1000, Millipore) and actin detected as a loading control with rabbit anti-actin (1:2000, Sigma). Secondary anti-rabbit-HRP (1:5000, Jackson Immunoresearch) was detected with EZ-ECL chemiluminescence (GeneFlow) and visualized with a LAS-3000 imager.

Expressed and secreted EspG from various strains were collected as previously described (Munera *et al*., 2010) and detected with rabbit anti-EspG (1:2000, a kind gift of A/Prof Neal Alto, UTSW) followed by anti-rabbit-HRP, or mouse anti-HA:HRP (1:5000). DnaK (1:5000, Stressgen) was assayed as a loading control.

### Statistical analysis

The mean ± SEM of multiple independent experiments are shown in all graphs. Statistical comparisons were produced by one- or two-way analysis of variance (anova) with Bonferroni's multiple comparison test (GraphPad PRISM, v5). *P < 0.05; **P < 0.01; ***P < 0.001; ****P < 0.0001.
